# Characteristics and determinants of high volume dispensing in long-term oral nutritional supplement users in primary care: a secondary analysis

**DOI:** 10.3399/BJGPO.2020.0131

**Published:** 2021-03-17

**Authors:** Patricia Dominguez Castro, Ciara Reynolds, Maria Gabriella Bizzaro, Sharon Kennelly, Barbara Clyne, Gerard Bury, Catriona Bradley, Karen Finnigan, Laura McCullagh, Celine Murrin, Carla Perrotta, Eileen R Gibney, Clare A Corish

**Affiliations:** 1 School of Public Health, Physiotherapy and Sports Science, University College Dublin, Dublin, Republic of Ireland; 2 UCD Institute of Food and Health, University College Dublin, Dublin, Republic of Ireland; 3 National Primary Care Division, Community Funded Schemes Service Improvement, Mountmellick Primary Care Building, Co. Laois, Republic of Ireland; 4 HRB Centre for Primary Care Research, Department of General Practice, Royal College of Surgeons in Ireland, Dublin, Republic of Ireland; 5 School of Medicine, University College Dublin, Belfield, Dublin, Republic of Ireland; 6 Royal College of Surgeons in Ireland, Dublin, Republic of Ireland; 7 Department of Pharmacology and Therapeutics, Trinity Centre for Health Sciences, St James’s Hospital, Dublin, Republic of Ireland; 8 School of Agriculture and Food Science, University College Dublin, Dublin, Republic of Ireland

**Keywords:** Malnutrition, Protein-energy malnutrition, oral nutritional supplements, polypharmacy, prescriptions, pharmacy, general practice, primary care

## Abstract

**Background:**

Oral nutritional supplements (ONS) are recommended for patients who are malnourished or at risk of malnutrition. Appropriate ONS prescribing requires regular monitoring to assess its continued requirement. Previous research identified long-term ONS prescriptions (>6 months) without review, with 70% of these influenced by social factors.

**Aim:**

To investigate the characteristics of long-term ONS users in Ireland and the determinants of larger volumes of ONS dispensing.

**Design & setting:**

Secondary analysis of anonymous dispensed pharmacy claims data of patients dispensed standard ONS for 12 consecutive months in 2018 (*n* = 912).

**Method:**

Factors showing significant (*P*<0.05) univariate associations with above the median consumption of ONS units were entered into a multivariable model.

**Results:**

Median age was 76 (range 18 to 101) years, with 66.9% of the sample being ≥65 years. Almost 70% of the samples were on polypharmacy (45.6%; ≥5 medications) or excessive polypharmacy (21.5%; ≥10 medications). Younger age and being on polypharmacy for drugs having an effect on the central nervous system (CNS) were significantly associated with being dispensed more ONS units in univariate and multivariate analysis. Those patients in the age range 18 to 44 were 2.5 fold more likely to be prescribed more ONS units (odds ratio [OR] 2.5; 95% confidence interval [CI] 1.5 to 4.3; *P*<0.001). Patients using CNS drugs or on CNS polypharmacy were more likely to be prescribed more ONS units (ORs 1.2 and 2.4; 95% CI 0.9 to 1.4 and 1.3 to 4.4 respectively; *P* = 0.029)*.*

**Conclusion:**

Older age and polypharmacy characterise long-term ONS users in this study. Younger age and CNS medication polypharmacy are predictors of more ONS units prescribed over a year*.*

## How this fits in

Malnutrition or undernutrition, arising from a deficiency of energy and protein intake, occurs commonly among community-dwelling individuals in developed countries. In the UK, it is estimated that approximately 3 million people are at risk of malnutrition, of which nearly half are aged over 65 years, the majority living in the community. Both in the UK and Ireland, it is estimated that 10% of the population requiring care from a GP are at risk of malnutrition. ONS are recommended for patients who are malnourished or at risk of malnutrition. Appropriate ONS prescribing requires regular monitoring to assess its continued requirement. Previous research has identified long-term ONS prescriptions (>6 months) without review, with 70% of these influenced by social factors. In Ireland, GPs are the main prescribers of ONS in primary care. They are also commonly the first point of contact for individuals in the community who are malnourished or at risk of malnutrition. Identifying the characteristics of long-term ONS users and the determinants of being in receipt of larger volumes of ONS can help to identify potential modifiable factors that would lead to evidence-based ONS prescribing and a reduction in unnecessary prescribing*.*

## Introduction

ONS are commercially manufactured products that provide macronutrients and micronutrients, and are used to supplement the diet of individuals who do not achieve their nutritional requirements through food alone. These products can be milk, juice, or soya-based, and are liquid, powder, or semi-solid in consistency.^1^ The UK National Institute for Health and Care Excellence (NICE) guidelines recommend the use of ONS for patients at risk of malnutrition or who are already malnourished.^2^ Malnutrition or undernutrition, arising from a deficiency of energy and protein intake, occurs commonly among community-dwelling individuals in developed countries, with population groups such as older people and people living with chronic conditions being at risk.^3–6^ Both in the UK and Ireland, it is estimated that 10% of the population requiring care from a GP is at risk of malnutrition.^7^

Malnutrition in the community is optimally treated by providing first-line dietary advice, a so-called ’food-first’ approach, in combination with ONS when necessary.^8,9^ Recent research investigating nutritional interventions in older adults at risk of malnutrition has indicated that ONS combined with dietary counselling is the most effective intervention, increasing both dietary intake and weight.^10^ Criteria for the appropriate prescribing of ONS have been proposed, which encompass nutritional screening and assessment to ascertain the need for ONS; investigation of the underlying causes of malnutrition; establishing desirable outcomes to be obtained from the nutritional intervention (for example, weight increase); and providing both dietary advice and continuous ONS monitoring to assess patient adherence and ongoing need.^11^

It is recommended that patients prescribed ONS should be reviewed every 3 months or, for those with longer term ONS requirements, every 6 months.^12^ Previous research in Ireland identified a large number of patients prescribed ONS for more than 6 months without review, with 70% of prescriptions influenced by social factors, such as living alone, and difficulties with cooking and shopping.^13^ The high cost for public health services associated with the prescribing of ONS has also been recognised in Ireland and the UK.^14,15^ Moreover, long term ONS prescribing can lead to patients’ over-reliance on these products to replace meals.^9^

The characteristics of long-term ONS users remain unexplored in the literature, and little is known about the determinants of higher volumes of ONS dispensed to individuals in the community. Therefore, this study aimed to investigate the characteristics of ONS long-term users residing in the community in Ireland, and the determinants of larger volumes of ONS dispensing among this population group.

## Method

### Study design and study population

This was a retrospective secondary analysis of anonymous dispensed pharmacy claims data for individual patients ≥18 years (*n* = 912) who were in receipt of ONS for 12 consecutive months in 2018 on the General Medical Services (GMS) Scheme in Ireland. The GMS is means tested for those <70 years, whereas all of those ≥70 years are eligible. Under the GMS scheme, persons are entitled to a medical card (MC) or a GP visit card (GPVC). MC holders receive free access to GP visits and prescribed medications, whereas GPVC holders are only entitled to free GP services. In 2018, 32.5% of the population of Ireland were eligible for the GMS Scheme; two million GMS cards were in circulation in 2018, with more than three quarters (76%) being MCs.^15–17^

### Database

A database of all prescriptions dispensed on the GMS Scheme is managed by the Health Service Executive Primary Care and Eligibility Reimbursement Service (PCERS). Within this database, demographic data for claimants and prescribers are available.

Patients were residents within three large population areas north, south, east, and west of Dublin (with surrounding areas). The areas examined contribute to 29.1% of the national population eligible for the GMS Scheme (Supplementary Table 3).

### Definition of study variables

This study aimed to describe the characteristics of long-term ONS users, and analyse the associations between demographic and medical factors (type and units of ONS, and type of non-ONS medications dispensed) in a year.

#### Demographic and ONS-related factors

The following information was analysed for the patient cohort; sex, age, living in residential care or independently, area of residence, GP practice area, number of individual ONS products dispensed, and ONS units dispensed in 2018, whether in receipt of ONS in 2017. Standard ONS were classified based on their energy and protein content (Supplementary Table 2). Individuals dispensed over the median number of annual ONS units (>660) for the study population were categorised as ‘high ONS users’.

The following information was analysed retrospectively for the patients’ GP of choice; sex, age, and practice area.

#### Non-ONS drugs

The number and type of non-ONS drugs dispensed were analysed for this cohort. General polypharmacy was defined as being dispensed ≥5 drugs and excessive polypharmacy as being dispensed ≥10 drugs.^18,19^ Ninety-three per cent were dispensed drugs working on the CNS, drugs working on the circulatory system, or both. As these categories represented the major medications in this cohort, drugs were further classified as CNS or circulatory, as per the 2019 Monthly Index of Medical Specialities medicine formulary in Ireland.^20^ The conditions treated by CNS and circulatory drugs in the study sample are presented in Supplementary Figure 2. For the purpose of the statistical analyses, CNS and circulatory drugs were coded as follows; no CNS/circulatory drugs, CNS/circulatory drugs (<5 drugs), and CNS/circulatory drug polypharmacy or excessive polypharmacy (≥5 drugs).

### Statistical analysis

Statistical analysis was carried out using SPSS (version 24.0). Discrete variables are presented as percentages, and continuous variables as medians and ranges. Sex differences were assessed using Mann–Whitney U and Kruskall–Wallis H non-parametric tests in the case of continuous variables, and using the χ^2^ test in the case of discrete variables.

Univariate analysis and multivariable analyses were performed with high ONS users as the dependent variable. Data were analysed using cross-tabulations and χ^2^ statistical test in the univariate analysis and binary logistic regression in the multivariable analysis, presenting adjusted OR and 95% CI.^21^ Independent variables were included in the logistic regression analysis if they were significant in the univariate analysis.^22^ Statistical significance was taken as a *P* value of <0.05.

## Results

### Sample characteristics

The median age of the study population was 76 years with around 67% of the sample being ≥65 years of age (Table 1). Women in the sample were older than men (82 versus 65 years old respectively*, P*<0.001) (Supplementary Table 1), and 18.2% of individuals were in residential care. Most patients were on one ONS product (90.0%), and the majority were in receipt of ONS in 2017 (96.2%). Men were dispensed more units of ONS in 2018 than women (684 units versus 576 units respectively, *P* = 0.017) (Supplementary Table 1). Over two-thirds of the sample either had polypharmacy or excessive polypharmacy. Women were dispensed more concomitant drugs compared to men (median 7 versus 5 drugs respectively, *P*<0.001) (Supplementary Table 1). In total, 75.4% were dispensed CNS drugs and 61.7% were dispensed circulatory drugs. The most commonly dispensed CNS drugs were those used to treat mood disorders and anxiety, and the most commonly dispensed circulatory drugs were those used to prevent myocardial infarction and to treat hypertension (Supplementary Figures 2 and 3, respectively).

**Table 1.** PCERS patients and GPs characteristics

Characteristic
Individuals on ONS, *n* = 912	%	Median (range)
**Sex**		
Male	43.5	
Female	56.5	
**Age**		76 (18 to 101)
≥18 to 44 years	11.3	
≥45 to 64 years	21.8	
≥65 years	66.9	
**Area**		
South/south east Dublin	16.0	
West/south west Dublin	41.4	
North/north west Dublin	42.5	
**Residential status**		
Living in residential care	18.2	
Living independently	81.7	
**Number of ONS products in 2018**		1 (1 to 4)
**Number of ONS units in 2018**		660 (12 to 3250)
**Patients on more than 1 ONS**	10.0	
**Patients on ONS previous year**	96.2	
**Number of medications**	6.0	(0 to 18)
**Polypharmacy**		
No polypharmacy (<5 drugs)	32.9	
Polypharmacy (≥5 drugs)	45.6	
Excessive polypharmacy (≥10)	21.5	
**Patient CNS drug** **dispensed** ^a^		
No CNS drug	24.8	
CNS drug (<5 drugs)	65.9	
CNS polypharmacy or excessive polypharmacy (≥5 drugs)	9.3	
**Patient circulatory drug dispensed** ^b^		
No circulatory drug	38.4	
Circulatory drug (<5 drugs)	42.7	
Circulatory polypharmacy or excessive polypharmacy (≥5 drugs)	19.0	
**GPs,** ***n*** **=** **405**		
**Sex**		
Male	59.0	
Female	41.0	
**Age**		53 (33 to 72)
≥18 to 44 years	23.2	
≥45 to 64 years	61.5	
≥65 years	15.3	
**Area**		
South/south east Dublin	23.2	
West/south west Dublin	35.1	
North/north west Dublin	39.3	
Other^c^	2.5	
**Number of patients on ONS**		2 (1 to 17)
**GPs** **with** **>** **2** **patients on ONS**	27.9	

^a^CNS medication include drugs to treat anxiety; insomnia; psychoses; mood disorders; obsessive compulsive disorder; nausea; vomiting; vertigo; epilepsy and seizures; Parkinson's; Alzheimer's; multiple sclerosis; attention deficit hyperactivity disorder, and narcolepsy. ^b^Circulatory medication include drugs to treat hypertension; oedema; heart failure; arrhythmias; angina; prevent myocardial infarction, stroke, and vascular events; hypercholesterolaemia; vascular diseases such as Raynaud's and pulmonary arterial hypertension; haemorrhage; anaemia and neutropenia. ^c^A minority of patients attended GPs with practices in different areas to their residential area.

CNS = central nervous system. GP = general practitioner. ONS = oral nutritional supplements. PCERS = Primary Care and Eligibility Reimbursement Service.

Most GPs were male and in the 45 to 64 years age category. The median number of long-term ONS users per GP was 2.0 (1 to 17). Just under one-third of GPs had more than two long-term ONS users under their care.

### Sample characteristics segregated by residential status

Table 2 shows that individuals living in residential care were older than those living independently (median 87 versus 71 years respectively, *P*<0.001). Individuals in residential care were also more likely to be dispensed more than one ONS product and be on polypharmacy or excessive polypharmacy (18.6% versus 8.2%, and 79.1% versus 64.5% respectively, both *P<*0.001).

**Table 2.** PCERS patients characteristics segregated by residential status

Characteristic	Median or %	*P* value
	Living in privatenursing home	Living independently	
**Ag** **e,** **years (***n*****=912**)**	87 (58 to 101)	71 (18 to 100)	*P*<0.001
**Sex**			
Male	11.3	88.7	
Female	23.7	76.3	*P*<0.001
**Area** **(** ***n*** **=** **912)**			
South/south east Dublin	17.4	15.7	
West/south west Dublin	47.3	40.1	
North/north west Dublin	35.3	44.2	*P*=0.110
**Number of ONS units in 2018** **(** ***n*** **=** **912** **)**	600 (30 to 3192)	672 (12 to 3250)	*P*=0.540
Patients on more than 1 ONS, *n* = 912	18.6	8.2	*P*<0.001
Patients on ONS previous year	92.8	96.9	*P*=0.023
**Number of medications**	7 (0 to 18)	6 (0 to 18)	*P*=0.021
**Polypharmacy**			
No polypharmacy (<5 drugs)	21.0	35.6	
Polypharmacy (≥5 drugs)	55.7	43.4	
Excessive polypharmacy (≥10)	23.4	21.1	*P*=0.001
**Patient CNS drug dispensed** ^a^			
No CNS drug	17.4	26.4	
CNS drug (<5 drugs)	74.3	64.0	
CNS polypharmacy or excessive polypharmacy (≥5 drugs)	8.4	9.5	*P*=0.032
**Patient** **c** **irculatory drug dispensed** ^b^			
No circulatory drug	33.5	39.5	
Circulatory drug (<5 drugs)	51.5	40.7	
Circulatory drug polypharmacy or excessive polypharmacy (≥5 drugs)	15.0	19.9	*P*=0.035

^a^CNS medication include drugs to treat anxiety; Insomnia; psychoses; mood disorders; obsessive compulsive disorder; nausea; vomiting; vertigo; epilepsy and seizures; Parkinson's; Alzheimer's; multiple sclerosis; attention deficit hyperactivity disorder, and narcolepsy. ^b^Circulatory medication include drugs to treat hypertension; oedema; heart failure; arrhythmias; angina; prevent myocardial infarction, stroke, and vascular events; hypercholesterolaemia; vascular diseases such as Raynaud's and pulmonary arterial hypertension; haemorrhage; anaemia and neutropenia.

CNS = central nervous system. ONS = oral nutritional supplements. PCERS = Primary Care and Eligibility Reimbursement Service.

### Characteristics of dispensed ONS

**Figure 1.**
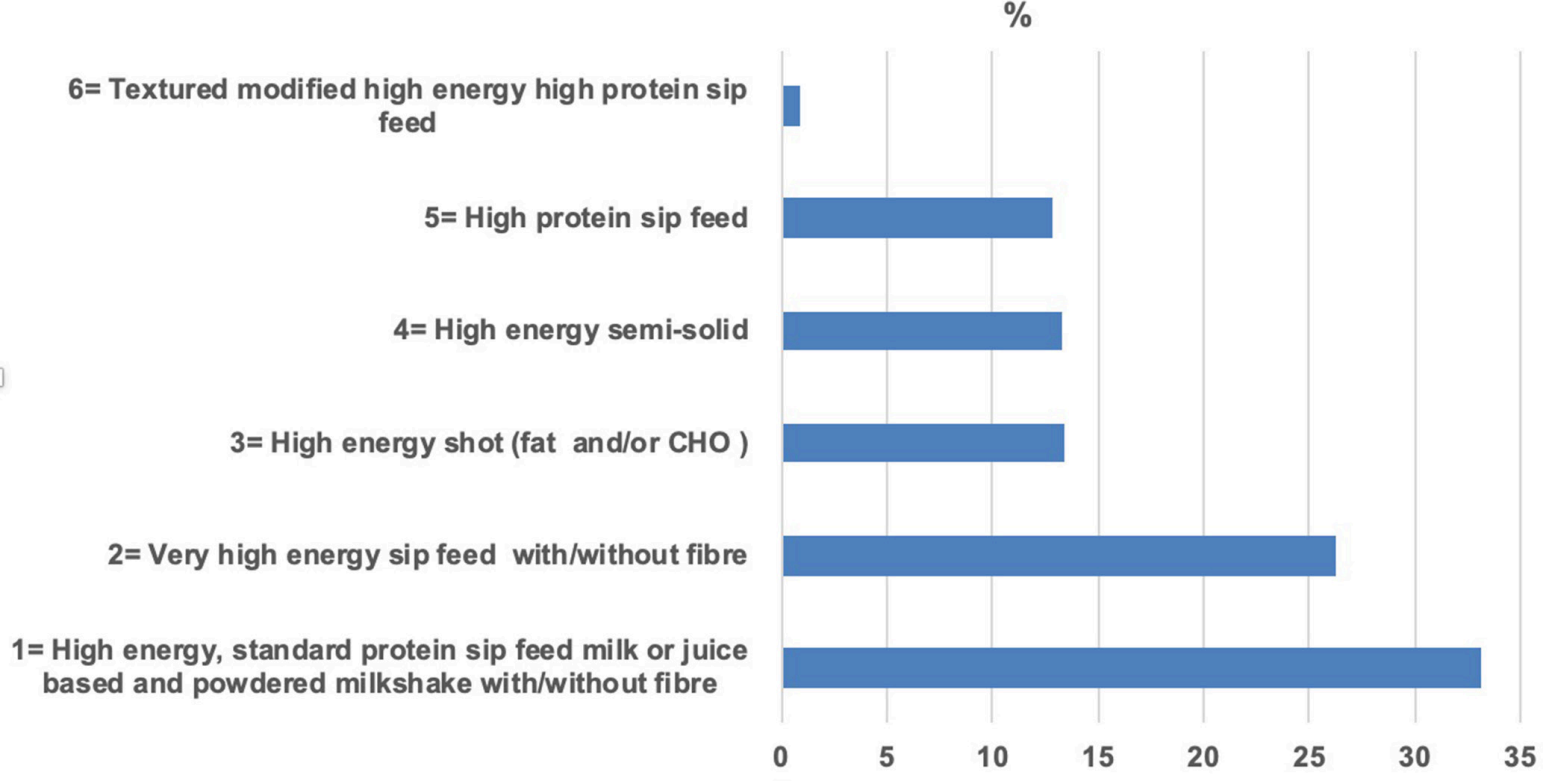
Percentage of oral nutritional supplements (ONS) product type dispensed to long-term ONS users (*n* = 912) as per Supplementary Table 2. CHO = Community Health Organisation.

The most commonly dispensed ONS were high-energy standard-protein sip feeds with or without fibre, followed by very high energy sip feeds with or without fibre. Together these represented 59.5% of all ONS products dispensed (Figure 1).

**Figure 2.**
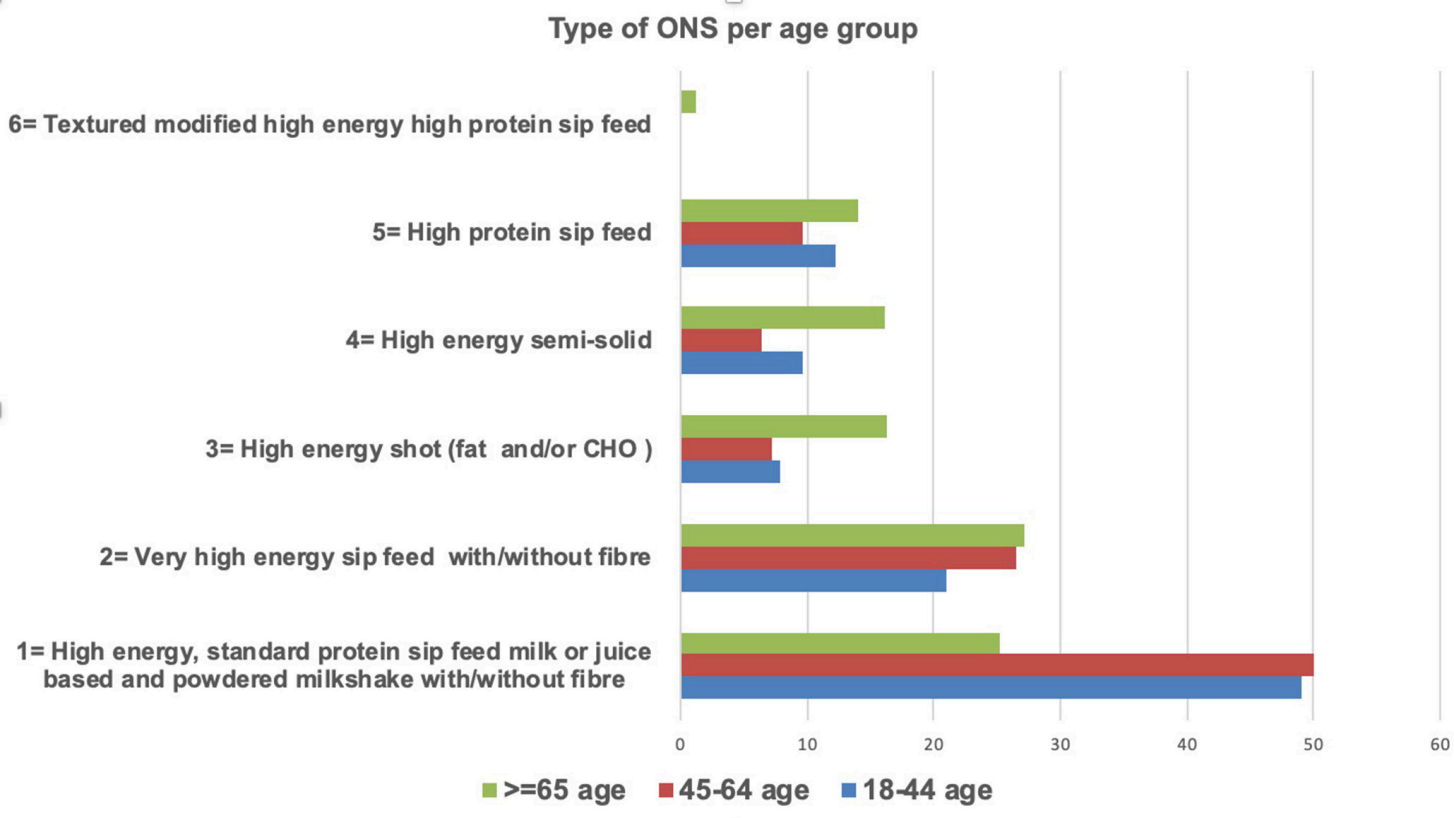
Percentage of oral nutritional supplements (ONS) product type dispensed to ONS long-term users (*n* = 912), segregated by patient age category. (*P*<0.001).

There were differences in ONS dispensing patterns within age categories; a larger proportion of patients aged 18 to 44 years and 45 to 64 years were in receipt of high-energy standard protein sip feeds compared to those aged ≥65 years (49.1%, 50%, and 25.2% respectively, *P*<0.001) (Figure 2).

### Univariate and multivariable analysis using median ONS units as the dependent variable

Univariate analysis showed that the variables associated with high ONS use were patient sex, patient age category, type of ONS, patient general polypharmacy, and patient CNS and circulatory drug prescription (Table 3). There was a higher proportion of patients with no polypharmacy (<5 drugs) in the high ONS user group compared to those with polypharmacy (>5 drugs) or excessive polypharmacy (>10 drugs) (57.3%, 46.6%, and 46.4% respectively, *P* = 0.009).

**Table 3.** Univariate analysis with characteristics of patients and GPs in the oral nutritional supplements (ONS) user and high ONS user groups

	<660 ONS units	≥660 ONS units	
C**haracteristic**	*n*	%	*n*	%	*P*-value
**Patients**					
**S** **ex**					
Male	181	45.6	216	54.4	
Female	274	53.2	236	46.8	0.023
**A** **r** **ea** **(** ***n*** **=** **912** **)**					
South/south east Dublin	84	57.5	62	42.5	
West/south west Dublin	184	48.7	194	51.3	
North/north west Dublin	187	48.2	201	51.8	0.130
**Residential status**					
Living in a private nursing home	90	53.9	77	46.1	
Living independently	365	49.0	380	51.0	0.270
**A** **ge category**					
≥18 to 44 years	34	33.7	67	66.3	
≥45 to 64 years	63	31.7	136	68.3	
≥65 years	358	58.7	252	41.3	<0.001
**Type of ONS** ^a^					
ONS type 1	239	45.7	284	54.3	
ONS type 2	208	64.0	117	36.0	<0.001
**G** **eneral** **p** **olypharmacy**					
No polypharmacy (<5 drugs)	128	42.7	172	57.3	
Polypharmacy (≥5 drugs)	222	53.4	194	46.6	
Excessive polypharmacy (≥10 drugs)	105	53.6	91	46.4	0.009
**CNS drug dispensed** ^b^					
No CNS drug	125	55.3	101	44.7	
CNS drug (<5 drugs)	302	50.2	299	49.8	
CNS polypharmacy or excessive polypharmacy (≥5 drugs)	28	32.9	57	67.1	0.002
**C** **irculatory drug dispensed** ^c^					
No circulatory drug	146	41.7	204	58.3	
Circulatory drug (<5 drugs)	207	53.2	182	46.8	
Circulatory polypharmacy or excessive polypharmacy (≥5 drugs)	102	59.0	71	41.0	<0.001
**GP** **s**					
**Sex**					
Male	294	49.5	300	50.5	
Female	161	50.6	157	49.4	0.781
**A** **ge category**					
≥18 to 44 years	113	46.3	131	53.7	
≥45 to 64 years	263	51.8	245	48.2	
≥65 years	79	49.4	81	50.6	0.370
**A** **rea**					
South/south east Dublin	102	54.0	87	46.0	
West/south west Dublin	161	49.1	167	50.9	
North/north west Dublin	182	47.6	200	52.4	
Other	10	76.9	3	23.1	0.116

^a^This variable only includes those patients who were on one ONS type, ONS type 1 includes the two most common type of ONS as per Figure 2 (high energy and very high energy standard protein sip feed with or without fibre), ONS type 2 includes the other ONS types represented in Figure 2 (high energy shots, high protein sip feed, high energy semi-solid, textured modified high energy/high protein sip feeds). ^b^CNS medication include drugs to treat anxiety; insomnia; psychoses; mood disorders; obsessive compulsive disorder; nausea; vomiting; vertigo; epilepsy and seizures; Parkinson's; Alzheimer's; multiple sclerosis; attention deficit hyperactivity disorder, and narcolepsy. ^c^Circulatory medication include drugs to treat hypertension; oedema; heart failure; arrhythmias; angina; prevent myocardial infarction, stroke, and vascular events; hypercholesterolaemia; vascular diseases such as Raynaud's and pulmonary arterial hypertension; haemorrhage; anaemia and neutropenia.

CNS = central nervous system. GP = general practitioner. ONS = oral nutritional supplements.

Multivariable analysis shows that patient age category, ONS type, and CNS drugs dispensed were significant predictors of high ONS use (Table 4). Compared to those individuals ≥65 years, individuals in the 18 to 44 years and 45 to 64 years age categories had higher odds of being high ONS users (ORs 2.5 and 2.5; 95% CI 1.5 to 4.3 and 1.7 to 3.7 respectively*, P*<0.001). Patients dispensed CNS drugs (<5) or on CNS polypharmacy or excessive polypharmacy had higher odds of being a high ONS user (ORs 1.2 and 2.4; 95% CI 0.9 to 1.4 and 1.3 to 4.4 respectively*, P*=0.029).

**Table 4.** Multivariable analysis with characteristics of patients significant in univariate analysis in the ONS users and high ONS users groups

Characteristics	Unadjusted^a^	Adjusted^b^
			95% CI			95% CI
			Lower	Upper			Lower	Upper
	OR	*P*			*P*	OR		
**Patient** **sex**								
Male	1.0^c^					1.0^c^		
Female	0.7	0.023	0.6	01.0	0.993	01.0	0.7	1.4
**Patient age category**								
≥18 to 44 years	2.9		1.9	4.5		2.5	1.5	4.3
≥45 to 64 years	3.1		2.2	4.3		2.5	1.7	3.7
≥65 years	1.0^c^	<0.001			<0.001	1.0^c^		
**Type of ONS** ^d^								
ONS type 1	1.0^c^					1.0^c^		
ONS type 2	0.5	<0.001	0.4	0.6	<0.001	0.6	0.4	0.8
**Patient** **p** **olypharmacy**								
No polypharmacy (<5 drugs)	1.0^c^					1.0^c^		
Polypharmacy (≥5 drugs)	0.7		0.5	0.9		0.8	0.6	1.2
Excessive polypharmacy (≥10)	0.6	0.010	0.5	0.9	0.550	0.8	0.5	1.3
**Patient CNS drug dispensed** ^e^								
No CNS drug	1.0^c^					1.0^c^		
CNS drug (<5 drugs)	1.2		0.9	1.7		1.2	0.9	1.8
CNS polypharmacy or excessive polypharmacy (≥5 drugs)	2.5	0.002	1.5	4.3	0.029	2.4	1.3	4.4
**Pati** **ent circulatory drug dispensed** ^f^								
No circulatory drug	1.0^c^					1.0^c^		
Circulatory drug (<5 drugs)	0.6		0.5	0.8		1.0	0.7	1.4
Circulatory polypharmacy or excessive polypharmacy (≥5 drugs)	0.5	<0.001	0.3	0.7	0.960	0.9	0.6	1.5

^a^Binary logistic regression carried out with each variable individually as predictor of dependent variables. ^b^Binary logistic regression carried out with full model adjusted for all variables as predictors of dependent variables. ^c^Indicates reference group d This variable only includes those patients who were on one ONS type, ONS type 1 includes the two most common type of ONS as per Figure 2 (high energy and very high energy standard protein sip feed with or without fibre), ONS type 2 includes the other ONS types represented in Figure 2 (high energy shots, high protein sip feed, high energy semi-solid, textured modified high energy/high protein sip feeds). ^e^CNS drugs include drugs to treat anxiety, insomnia, psychoses, mood disorders, obsessive compulsive disorder, nausea, vomiting, vertigo, epilepsy and seizures, Parkinson's, Alzheimer's, multiple sclerosis, attention deficit hyperactivity disorder, and narcolepsy. ^f^Circulatory medication include drugs to treat hypertension; oedema; heart failure; arrhythmias; angina; prevent myocardial infarction, stroke, and vascular events; hypercholesterolaemia; vascular diseases such as Raynaud's and pulmonary arterial hypertension; haemorrhage; anaemia and neutropenia.

CI = confidence interval. CNS = central nervous system. ONS = oral nutritional supplements. OR = odds ratio.

## Discussion

### Summary

In this study, long-term ONS users in the community setting were characterised by female sex, older age, and polypharmacy. Sex differences existed among these ONS users, with females being older than males. Interestingly, when looking at the determinants of higher dispensing of ONS units, patients’ younger age, and CNS drug prescription were predictors of more ONS units dispensed over a year.

### Strengths and limitations

A strength of this study is that, to the authors' knowledge, it is the first to investigate the characteristics of long-term ONS users and the determinants of higher prescribing of ONS units in the community setting. Despite the population being limited to three areas in Dublin, the database represents almost one-third of all patients living in Ireland who are eligible nationally for the GMS Scheme and gives insight into urban- and suburban-dwelling individuals who are living independently or in residential care.

A limitation of the study is the lack of other contextual variables needed to explore in more detail the characteristics of this sample, such as presence of chronic conditions, access to dietetic services, level of social support, and hospitalisation. Moreover, due to the eligibility criteria, the GMS scheme over-represents socioeconomically deprived individuals, and the population in this study may be affected by multimorbidity and depression to a greater extent than a less deprived sample of a similar age. Furthermore, the database used only represented those individuals in the GMS who are MC holders, as GPVC holders are not entitled to free or reduced cost dispensed medications. The polypharmacy variable was constructed from a list of drugs prescribed for each patient for which pharmacy claims were presented for payment. The authors do not have information on the duration of prescription or whether these were repeat prescriptions. Moreover, the data contained in the PCERS database are for dispensed, and not prescribed, ONS, therefore, it is impossible to ascertain whether patients are taking their ONS as prescribed or the adequacy of the ONS prescription.

### Comparison with existing literature

To the authors' knowledge, there are no previous studies that have characterised adult long-term ONS users in the community. It is interesting that different studies investigating the factors associated with and the determinants of malnutrition in older adults have identified older age, female sex, and polypharmacy with increased risk of malnutrition.^23,24^ The association between age and malnutrition is unclear and could be mediated instead by the progressive deterioration of health and functional ability associated with ageing, also known as frailty.^24^

Although female sex has been associated with a higher risk of developing malnutrition, this finding was not consistently observed in systematic reviews.^24,25^ It may be that the increased risk of developing malnutrition in females is linked to more disabilities and greater functional decline compared with males.^24,26^ The cohort of long-term ONS users in this study was predominantly female, and women were dispensed significantly more concomitant drugs than men, possibly indicating that they are frailer.

Contradictory results have also been reported on the association between polypharmacy and malnutrition.^24^ Polypharmacy could increase the risk of malnutrition due to its side effects, including poor appetite, among others.^24^ More than 66% of this study's sample of long-term ONS users were either on polypharmacy or excessive polypharmacy; however, a larger percentage of those who were not on polypharmacy or excessive polypharmacy were in receipt of more ONS units than the median. This could suggest that those on polypharmacy or excessive polypharmacy were a frailer cohort, with lower levels of energy intake, supported by the fact that a larger proportion of older people (≥65 years) had polypharmacy or excessive polypharmacy compared to the younger age groups (Supplementary Figure 1).

Different patterns of ONS prescribing were observed in the sample of long-term ONS users when categorised by age, with a larger percentage of those between 18 to 64 years prescribed high-energy standard-protein sip feeds, and a larger percentage of those ≥65 years being prescribed one of the other ONS categories. This may be explained by the deficits in the ability of older people to meet their energy and protein requirements.^27^ Interestingly, although it could be expected that more ONS units would be prescribed for those in residential care, being a frailer cohort, there was no difference between the units dispensed for this cohort and those living independently in the community. A more supportive environment with foods for individuals in residential care could partially explain the equal units dispensed in both settings.^28^

### Implications for research and practice

The population of long-term ONS users in Ireland is characterised by older age. Decreased functional ability during ageing can negatively impact on the ability to shop and cook meals, which, in turn, detrimentally influences nutritional status. In fact, previous research in Ireland reported that social factors, such as difficulties with cooking and shopping, provide the rationale for prescribing ONS in 70% of cases.^13^

Furthermore, in this cohort, CNS medication polypharmacy was a predictor of more ONS units prescribed over a year in long-term users. Little is known about mental health and ONS prescription; however, mental health problems, particularly anxiety and depression, commonly occur in older people, who are vulnerable to develop malnutrition.^23,29^ Thus, mental health problems could be directly associated with the development of malnutrition.^23,29,30^ A Swedish study in 2011 reported that mental health symptoms were strongly associated with the risk of malnutrition in community-dwelling older people.^29^ A bidirectional association between mental health and malnutrition has been suggested. Depression can cause a decrease in appetite; however, malnutrition is linked to micronutrient deficiencies which can negatively impact on mental health.^31,32^ It is interesting to observe how in this study, younger age was also a predictor of higher dispensing of ONS units; this finding deserves further exploration, if possible alongside information relating to chronic health conditions and hospitalisation, as this could indicate social prescribing of ONS to young adults with mental health issues and chaotic lifestyles.^33^

Finally, the majority of this cohort was already in receipt of ONS in 2017 indicating that, once the ONS prescription is commenced in patients with the characteristics described in this study, its discontinuation is unlikely to happen. Oral intake of a high-calorie, high-protein diet should be promoted whenever possible in patients with or at risk of malnutrition, with ONS being used as an adjunct to the diet for a limited period when necessary.^12^ It has been previously reported that primary healthcare professionals occasionally prescribe ONS for patients with or at risk malnutrition in the community when food modification has not been possible due to social factors.^34,35^ Also, better compliance with ONS has been reported by individuals living alone and with difficulties cooking and shopping. Further research should explore whether social support for long-term ONS users would reduce their need to be prescribed ONS. It would also be interesting to compare the characteristics of individuals on long-term ONS but who are not included on the GMS scheme, as with this study's sample. GPs may benefit from collaboration with other healthcare professionals with expertise in nutrition, such as community dietitians, in the treatment of patients with malnutrition.^9^ Box 1 provides links to guidelines on ONS prescribing and reviewing.

Box 1 **Links for guidance on ONS prescribing/reviewing**

- Prescribing Pathway for the Initiation and Renewal of Standard Oral Nutritional Supplements (ONS) for Adults Living in the Community https://www.hse.ie/eng/about/who/cspd/ncps/medicines-management/oral-nutritional-supplements/prescribing%20pathway%20and%20list%20.pdf
- Managing malnutrition in the community. Pathway for using oral nutritional supplements (ONS) in the management of malnutrition. https://www.malnutritionpathway.co.uk/library/ons_pathway.pdf
